# Antimicrobial Peptide Analogs From Scorpions: Modifications and Structure-Activity

**DOI:** 10.3389/fmolb.2022.887763

**Published:** 2022-05-26

**Authors:** Bruno Amorim-Carmo, Adriana M. S. Parente, Eden S. Souza, Arnóbio A. Silva-Junior, Renata M. Araújo, Matheus F. Fernandes-Pedrosa

**Affiliations:** ^1^ Laboratory of Pharmaceutical Technology and Biotechnology, Pharmacy Department, Federal University of Rio Grande do North, Natal, Brazil; ^2^ School of Biomolecular and Biomedical Sciences, University College Dublin, Dublin, Ireland

**Keywords:** antimicrobial activity, structural modification, scorpion peptides, hemolytic activity, analog peptides

## Abstract

The rapid development of multidrug-resistant pathogens against conventional antibiotics is a global public health problem. The irrational use of antibiotics has promoted therapeutic limitations against different infections, making research of new molecules that can be applied to treat infections necessary. Antimicrobial peptides (AMPs) are a class of promising antibiotic molecules as they present broad action spectrum, potent activity, and do not easily induce resistance. Several AMPs from scorpion venoms have been described as a potential source for the development of new drugs; however, some limitations to their application are also observed. Here, we describe strategies used in several approaches to optimize scorpion AMPs, addressing their primary sequence, biotechnological potential, and characteristics that should be considered when developing an AMP derived from scorpion venoms. In addition, this review may contribute towards improving the understanding of rationally designing new molecules, targeting functional AMPs that may have a therapeutic application.

## Introduction

Antimicrobial peptides (AMPs) are constitutively expressed small bioactive molecules (Peters et al., 2010; Soliman et al., 2013). However, studies indicate that proteolytic cleavage of functional proteins can result in AMPs (Lyapina et al., 2019). AMPs have been identified in a variety of life forms, such as unicellular microorganisms, invertebrates, plants, amphibians, birds, fish, and mammals, including humans, where they act as part of the innate immune system against pathogenic microorganisms (Gopal et al., 2013; Almaaytah and Albalas, 2014). Altogether, diversity of AMPs and their presence in a variety of organisms indicate their relevance for immune defense (Brogden and Brogden, 2011; Fjell et al., 2012).

AMPs can also show activity against tumor cells (Crack et al., 2012), virus (Luplertlop et al., 2011), and protozoa (Torrent et al., 2012), and for this reason, they are often classified as multifunctional peptides (Zeng et al., 2013). These activities are not necessarily excluding, and AMPs might also act synergistically in more than one activity (Conlon et al., 2010; Gong et al., 2010; Zeng et al., 2013).

Although invertebrates lack adaptative immunity, they can rely on an effective innate response, which is not as basic as once thought (Divangahi et al., 2021). AMPs are key molecules in the defense mechanism of invertebrates (Wu et al., 2021). Within arthropods, insects are the most studied class, and the AMP diversity in this class includes the AMP families cecropins, defensins (Brady et al., 2019), and gloverins (Wang et al., 2019), among others (Wu et al., 2018; Buonocore et al., 2021).

Arthropods also include scorpions, from which several bioactive molecules have been isolated from the venom glands. AMPs are one of these molecules and aid in the protection of the gland against infections caused by saprophytic organisms and facilitate the activity of neurotoxins (Hernández-Aponte et al., 2011; Ahmadi et al., 2020; Shahzadi et al., 2021). The diversity of AMPs isolated from scorpions comprises families present in a variety of life forms, such as defensins (Cheng et al., 2020), and AMPs that were first known to be present in scorpion species, such as scorpine from *Pandinus imperator* (Conde et al., 2000) and Heterin-2 from *Heterometrus spinifer* (Wu S. et al., 2014). Scorpion AMPs were classified elsewhere as proline- and glycine-rich AMPs, AMPs with cysteine that create disulfide bridges, and α-helical AMPs without cysteine (Harrison et al., 2014). Although α-helical AMPs are more common, scorpion venom may also contain AMPs with disulfide bridges, as in the case of HS-1 that has three of these interactions (Uawonggul et al., 2007). Cationic AMPs without disulfide bridges in scorpions have already been demonstrated to have multifunctional activity, for example, antifungal, antiviral, antiparasitic, and antiproliferative (Miyashita et al., 2010; Almaaytah et al., 2014; Du et al., 2014; Erdeş et al., 2014). The main mechanism of action reported is the formation of pores in the cell membrane of the pathogen, which reduces the ability of these microorganisms to develop resistance (Almaaytah and Albalas, 2014; Parachin and Franco, 2014; Ortiz et al., 2015).

The first AMP reported from scorpion venom, named Hadrurin, was isolated from the species *Hadrurus aztecus* in 2000 (Torres-Larios et al., 2000; Almaaytah et al., 2012); ever since, several AMPs from different scorpion species have been isolated and characterized (Harrison et al., 2014). Scorpion venom AMPs usually present a random structure in aqueous media and a predominant helix structure in hydrophobic media. This ability to acquire different conformations in different media may be related to their activity in cell membranes (Li et al., 2014; Daniele-Silva et al., 2016; Luna-Ramirez et al., 2017). Moreover, AMPs from distinct scorpion species have been observed to present hemolytic and cytotoxic effects against eukaryotic cell lines, and high susceptibility to the activity of proteolytic enzymes, which is a hindrance to their therapeutic application (Yeaman and Yount, 2003; Zeng et al., 2012; Almaaytah and Albalas, 2014).

Nevertheless, there is still great interest in the development of AMPs as a potential class of antimicrobial agents, due to their high activity *in vitro* with a broad action spectrum (Almaaytah et al., 2012). Thereby, various structural determinants are responsible for the antimicrobial and cytolytic activities of these peptides. Studies demonstrate that the increment in the α-helix conformation, cationic character, hydrophobicity, and the hydrophobic moment of a native AMP can enhance its antibiotic action spectrum (Wang et al., 2008; Zhao et al., 2009; Nguyen et al., 2011). C-terminal amidation is another important AMP modification as it can increase resistance to proteolysis and stability of the amphipathic helices (Sforça et al., 2004). The substitutions of amino acids in the short peptide chain have shown alterations in the structural conformation and, therefore, can improve the characteristics of native peptides, increasing their biological activity and stability and reducing their toxicity. Therefore, modification of the chemical composition of AMPs is a powerful and promising tool to increase their biotechnological potential and to develop more bioactive and less toxic antimicrobial molecules (Nguyen et al., 2011; Fjell et al., 2012; Parente et al., 2018).

A clearer understanding of the influence of these characteristics on peptide structures can help develop new strategies that will facilitate the design of pharmacological agents that will improve or optimize their therapeutic potential, as well as reduce the rate of microbial resistance acquired from AMPs. Thus, this review aims to address the AMPs found in scorpion venom and the most common modifications used to increase their antimicrobial activity and modulate their toxicity to human cells, in addition to the possible application of scorpion AMPs as an anti-infective therapeutic agent.

## Physicochemical Properties That Influence the Antimicrobial Activities of Antimicrobial Peptides

### Peptide Sequence and Conformation

Scorpion AMPs are small amino acid chains, usually ranging from about 10 to 25 amino acids, and it has been suggested that sequence length may be of significance to their activity. The short size makes AMPs more attractive from the chemical synthesis point of view, as it is less costly to synthesize small molecules (Li and Brimble, 2019). Therefore, small peptides are more advantageous for large-scale chemical synthesis.

The sequence size is also important to their defense function, since, when compared with bigger robust proteins, they can insert in the cytoplasmic membrane efficiently. Furthermore, a study using artificial intelligence 100,000 peptides of which 200 were synthetized and tested; the results showed that a peptide shorter than usual, containing nine amino-acid residues, presented minimum inhibitory concentration (MIC) lower than 1 µM (Cherkasov et al., 2009). Other studies using antimicrobial peptides derived from other animals also showed that shortened peptides produced better antimicrobial activity (Solstad et al., 2020; Fuscaldi et al., 2021).

Although scorpion AMPs have a highly diverse amino acid sequence, hydrophobic and basic residues are found in most peptides. The presence of such residues results in similar physicochemical properties among AMPs. In addition to the observed heterogeneity, these peptides present similar physicochemical properties, such as amphipathicity and positive net charge, which are important to their interactions with microbial membranes (Nguyen et al., 2011; Leite et al., 2015). One common posttranslational modification used in AMPs is C-terminal amidation, which is highly important not only to protect these peptides from proteolysis but also because it plays a role in their antimicrobial activity by stabilizing the peptide–membrane interaction (Dennison et al., 2015; Mura et al., 2016).

**FIGURE 1:**
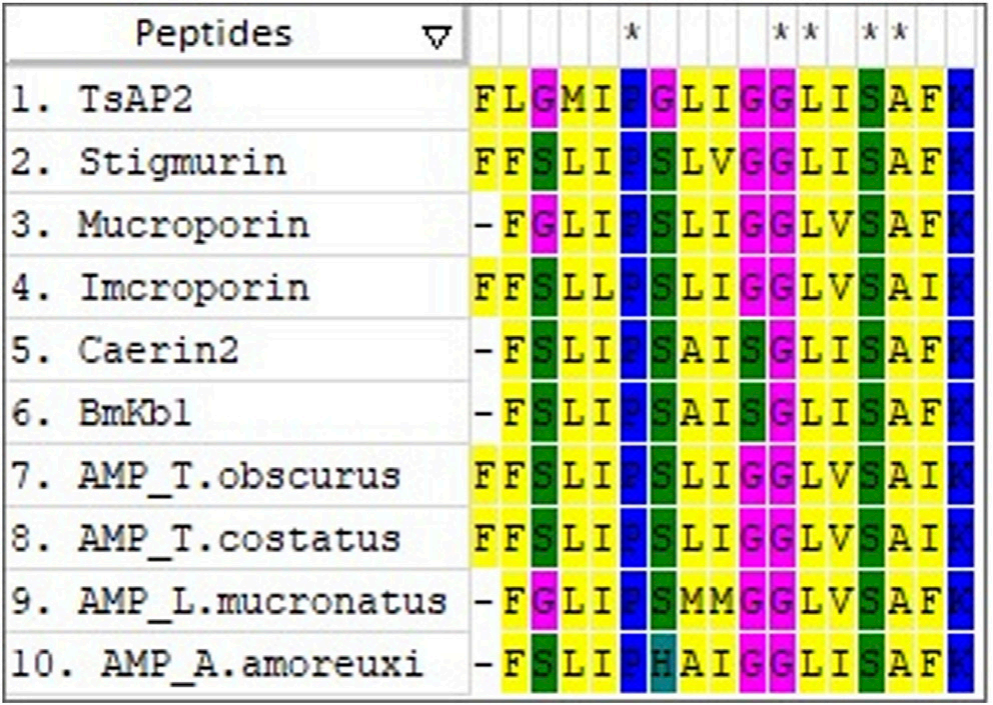
Sequence alignment of 10 scorpion antimicrobial peptides. *Conserved residues.

Conserved amino acid residues found in different peptides play a similar role. In Figure 1 we present a sequence alignment between AMPs from different scorpion species, in which conserved residues can be observed throughout the sequence. At the center of these sequences, there is a G-L-I/V-S-A domain. These conserved amino acids may be important for their activity, or even for the maintenance of the peptide structure.

Scorpion AMPs present a more flexible structure as most of them lack cysteine and disulfide bonds. This feature allows such AMPs to alter their conformation according to the environment. In hydrophilic solvents, scorpion AMPs present random conformation, but when in hydrophobic solvents, they show an α-helical conformation. This behavior has been confirmed through *in vitro* and *in silico* experiments (Melo et al., 2015; Parente et al., 2018). This conformation change grants that when AMPs are in an aqueous intercellular environment, they present random conformation, thus being inactive, but when in contact with the membrane hydrophobic environment, they assume an α-helical conformation and so insert in the membrane (Nguyen et al., 2011; Almaaytah and Albalas, 2014). Thus, both peptide sequence and conformation are important for AMPs’ insertion in the membrane and activity. However, due to limited studies, information on the specific impact of each amino acid on the peptide action mechanism is still scarce.

### C-Terminal Amidation

Peptides can be rapidly hydrolyzed when in contact with proteases presented in our organism or proteases produced by bacteria (Moncla et al., 2011). Hence, they can show lower oral and plasmatic activity (Christophersen et al., 2014). This has boosted studies that aim, through the rational amino-acid deletion, addition, or replacement, the development of biologically active analogs with physicochemical properties capable of enhancing their potentiality and improving their stability against proteases (Kumar and Bhalla, 2005; Nešuta et al., 2017).

In the vast group of AMPs extracted from various organisms, the importance of C-terminal amidation is highlighted, and most bioactive native peptides are known to present it, establishing that this can be an important characteristic for their activity. Furthermore, several studies have evaluated the effect of C-terminal amidation on AMPs’ stability, efficiency, and toxicity (Sforça et al., 2004). It was shown that amidated peptides were more resistant to protease activity and that amidation stabilizes α-helix formation (Brinckerhoff et al., 1999; Dennison and Phoenix, 2011).

However, one study analyzed several amidated and non-amidated antimicrobial peptides and observed that amidation of the C-terminal region does indeed have a variable effect on the peptide structure, as some of these peptides lost their activity, while others maintained their antimicrobial activity in the absence of amidation (Strandberg et al., 2007). The same study showed that a peptide’s toxicity to prokaryotic and eukaryotic cells might not be affected by the presence or absence of the C-terminal amidation, indicating that it has a variable effect on the peptide sequence (Strandberg et al., 2007). Dennison et al. (2009) concluded in their study that C-terminal amidation of defense peptides has a variable effect on their antimicrobial activity and no clear effect on their selectivity for these cell types. Therefore, C-terminal amidation needs to be considered based mainly on the structure of the native peptide (presence or absence of C-terminal amidation) used as a prototype when designing a new antimicrobial agent.

### Amphipathicity

The amphipathic (A) aspect of AMPs with α-helical conformation is directly dependent on the amino acid sequence (Dennison et al., 2005; Wiradharma et al., 2011; Zhang et al., 2016; Brul, 2018; Riahifard et al., 2018). Hydrophobic amino acids are periodically distributed in amphipathic helices, and a simple replacement of arginine by lysine can boost activity without increasing the toxicity to the host (Giménez-Andrés et al., 2018; Luo et al., 2021).

Initially, hydrophobic residues insert in the membrane and interact through hydrophobic forces, causing membrane permeabilization (Shai, 1999; Karmakar et al., 2019). Insertion of the peptide into the membrane process starts with the attachment of the amphipathic cationic helix to the membrane in a flat orientation. The membrane–AMP interaction occurs with the AMP hydrophobic face interacting with the membrane and the polar face of the peptide interacting with the solvent. Then, the complex membrane–AMP reaches an equilibrium state prior to the pore-forming activity (Gagnon et al., 2017).

**FIGURE 2:**
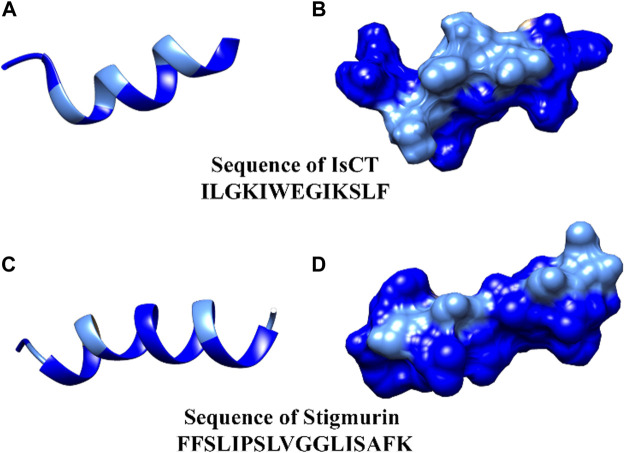
Theoretical tridimensional structure using UCSF Chimera software (Pettersen et al., 2004) for the peptides IsCT **(A)** and Stigmurin **(C)**. In **(B,D)** peptides IsCT and Stigmurin in electrostatic surface, respectively. Light blue and dark blue represent hydrophilic and hydrophobic regions, respectively.

To better illustrate the amphipathicity of α-helical AMPs, two AMP structures from scorpion species were chosen: IsCT, a peptide present in *Opisthacanthus madagascariensis*, and Stigmurin, isolated from *Tityus stigmurus* (Figure 2). It is possible to observe the amphipathic character of these structures through the hydrophobic and hydrophilic regions well distributed along the peptide chain. It is noticeable that Stigmurin exhibits greater hydrophobicity than IsCT (Dai et al., 2001; Daniele-Silva et al., 2021).

Although amphipathicity is very important to the antimicrobial activity, its increment does not seem to increase the antibacterial activity (Yan et al., 2003). Tossi et al. (2000) also observed that the antimicrobial activity of AMPs is little affected by a change in amphipathicity. On the other hand, the superficial amphipathicity of peptides is associated with cell type selectivity, as it was observed by Wiradharma and collaborators (2011). The authors found that the synthetic peptides (FFRR)_3_, (LLRR)_3_, and (LLKK)_3_ showed stronger antimicrobial activity and lower toxicity against red blood cells when compared with another peptide tested (AARR)_3_. The authors attribute the lack of activity of (AARR)_3_ to poorer amphipathicity in this peptide, which was caused by high hydrophilicity of alanine residues. Other peptides designed with two and four repeats (i.e., (FFRR)_2_), showed lower antimicrobial activity and higher toxicity. This indicates that the size and amino acid composition of the amphipathic helix may be modulated to obtain greater efficacy. Although amphipathic cationic helices are widely common among AMPs, there are many reports of AMPs structured as β-sheets, especially designed peptides. As an example, the synthetic peptide (KIGAKI)_3_-NH_2_, which was designed to have amphipathic features and fold as a β-sheet, exhibited higher antimicrobial activity and lower hemolytic activity than the α-helical peptides tested by the authors (Blazyk et al., 2001). β-Sheets are reported in AMPs from the scorpion *Mesobuthus martensii*, for example, CSαβ defensin and HAP-1 from *Mesobuthus martensii* (Lang et al., 2017; Shi et al., 2018). However, synthesis of α-helical peptides is still preferred due to its lower cost. β-Sheet conformations exhibit disulfide bridges to stabilize their structure, thus increasing the synthesis cost (Groß et al., 2016).

### Hydrophobicity

As aforementioned, AMPs have both acidic and basic amino acids; therefore, they present an amphipathic character that gives them the ability to solubilize in both hydrophobic and hydrophilic solvents. These peptides present particularly high hydrophobicity (H), which is important for their insertion into the cytoplasmic membrane through hydrophobic interactions (Toke, 2005).

Nevertheless, studies have demonstrated that high levels of hydrophobicity can lead to peptide aggregation and precipitation; hence, increased hydrophobicity can decrease the peptide’s antimicrobial activity (Zou et al., 2018). Studies show a much more direct correlation between hydrophobicity and hemolytic activity (Chang et al., 2017; Parente et al., 2018). Hydrophobicity changes caused differences in the peptide’s ability to insert into electrically neutral membranes (Dathe et al., 1996; Wieprecht et al., 1997).

Hydrophobicity, as already mentioned, is important for the interaction of AMPs with cytoplasmic membranes. So, it is likely that an increased hydrophobicity enables the peptide interaction with membranes with no selectivity, which can lead to toxicity towards mammalian cells. However, there are controversies regarding the correlation between hydrophobicity and hemolysis. In a study with two analog peptides from Stigmurin, an AMP from the scorpion *T. stigmurus*, the native peptide, which presented higher hydrophobicity than the analogs, showed no hemolysis, but the analog peptides showed hemolysis rate up to 30% at the tested concentrations (Melo et al., 2015; Parente et al., 2018) (Tables 1, 2). Thereby, hydrophobicity is an important peptide feature related to its activity and toxicity, although the increased antimicrobial activity caused by the increment in hydrophobicity may be related to the chemical characteristics and composition of AMPs.

**TABLE 1:** Physicochemical characteristics of native antimicrobial peptides and scorpion analogs.

Scorpion	Native peptide	H	µH	Z	Analog peptide^a^	Residue substitution	H	µH	Z	References
*Tityus stigmurus*	**Stigmurin** FFSLIPSLVGGLISAFK-NH_2_	0.89^b^	0.57	+2	**StigA6** FFSLIP **K** LV **K** GLISAFK-NH_2_	Ser and Gly by Lys	0.78^b^	0.66	+3	Parente et al. (2018)
					**StigA16** FF **K** LIP **K** LV **K** GLISAFK-NH_2_		0.72^b^	0.72	+4	
					**StigA25** FFSLIPSLV **KK** LI **K** AFK-NH_2_	Ser and Gly by Lys	0.73	0.70	+5	Amorim-Carmo et al. (2019)
					**StigA31** FF **K** LIP **K** LV **KK** LI **K** AFK-NH_2_	Ser and Gly by Lys	0.61	0.80	+7	
*Vaejovis mexicanus smithi*	**VmCT1** FLGALWNVAKSVF	0.82	0.58	+2	**[K]** ^**3**^ **-VmCT1-NH**_**2**_ FL **K** ALWNVAKSVF	Gly by Lys	0.74	0.64	+3	Pedron et al. (2017)
					**[K]** ^**7**^ **-VmCT1-NH**_**2**_ FLGALW **K** VAKSVF	Asn by Lys	0.79	0.60	+3	
					**[K]** ^**11**^ **-VmCT1-NH**_**2**_ FLGALWNVAK **K** VF	Ser by Lys	0.75	0.63	+3	
					**[E]** ^**4**^ **-VmCT1-NH**_**2**_ FLG **E** LWNVAKSVF	Ala by Glu	0.75	0.61	+1	
					**[E]** ^**7**^ **-VmCT1-NH**_**2**_ FLGALW **E** VAKSVF	Asn by Glu	0.82	0.58	+1	
					**[W]** ^**9**^ **-VmCT1-NH**_**2**_ FLGALWNV **W** KSVF	Ala by Trp	0.97	0.72	+2	
					**[E]** ^**4**^ **[W]** ^**9**^ **-VmCT1-NH**_**2**_ FLG **E** LWNV **W** KSVF	Ala by Glu and Trp	0.90	0.76	+1	
*Buthus martensii* Karsh	**BmKn2** FIGAIARLLSKIF-NH_2_	0.84^b^	0.76^b^	+2^b^	**BmKn2K7** FIGAIA **K** LL **K** KIF-NH_2_	Arg and Ser by Lys	0.77^b^	0.80^b^	+3^b^	de la Salud Bea et al. (2015)
					**BmKn2L1** FIGA **L** ARL **L** SKIF-NH_2_	Arg by Leu	0.83^b^	0.75^b^	+2^b^	
					**BmKn2V1** FIGA **V** ARL **V** SKIF-NH_2_	Ile and Leu by Val	0.76^b^	0.68^b^	+2^b^	
					**BmKn2A1** FIGA **A** ARL **A** SKIF-NH_2_	Ile and Leu by Ala	0.62^b^	0.55^b^	+2^b^	
	**BmKn1** FIGAVAGLLSKIF-NH_2_	0.87^b^	0.63^b^	+1^b^	**BmKn1-6Lys** FIGAV **K** GLLSKIF-NH_2_	Ala by Lys	0.77^b^	0.64^b^	+2^b^	
					**BmKn1L2K2** F **K** GA **L** AGL **L** SK **K** F-NH_2_	Ile and Val by Leu and Lys	0.48^b^	0.35^b^	+3^b^	
*Androctonus amoeruxi*	**AamAP1** FLFSLIPHAIGGLISAFK-NH_2_	0.90	0.43	+1	**AamAP-S1** FLFSLIP **K** AIGGLISAFK-NH_2_	His by Lys	0.84^b^	0.49^b^	+2^b^	Almaaytah et al. (2012)
					**A3** FLFSLI **RK** AIGGLISAFK	Pro and His by Arg and Lys	0.74	0.51	+3	Almaaytah et al. (2018)
					**AamAP1-Lysine** FLF **K** LIP **K** AI **KK** LIS **K** FK	Ser, His, Gly, and Ala by Lys	0.61	0.61	+6	Almaaytah et al. (2014)
*Opithancatus madagascariensis*	**IsCT1** ILGKIWEGIKSLF-NH_2_	0.78^b^	0.77^b^	+1^b^	**IsCT1A1** ILGK **A** WEG **A** KSLF-NH_2_	Ile by Lys	0.55^b^	0.56^b^	+1^b^	de la Salud Bea et al. (2017)
					**IsCT1V1** ILGK **V** WEG **V** KSLF-NH_2_	Ile by Val	0.69^b^	0.69^b^	+1^b^	
					**IsCT1L1** ILGK **L** WEG **L** KSLF-NH_2_	Ile by Leu	0.76^b^	0.76^b^	+1^b^	
					**IsCT1K7** ILGKIW **K** GIKSLF-NH_2_	Glu by Lys	0.75^b^	0.80^b^	+3^b^	
					**IsCT1E7** ILGKIWEGI **E** SLF-NH_2_	Lys by Glu	0.71^b^	0.76^b^	−1^b^	
	**IsCT2** IFGAIWNGIKSLF	0.89^b^	0.71^b^	+1^b^	**IsCT2A1** ILGA **A** WNG **A** KSLF-NH_2_	Ile by Ala	0.65^b^	0.49^b^	+1^b^	
					**IsCT2V1** ILGA **V** WNG **V** KSLF-NH_2_	Ile by Val	0.79^b^	0.62^b^	+1^b^	
*Tityus serrulatus*	**TsAP-1** FLSLIPSLVGGSISAFK-NH_2_	0.78^b^	0.43	+2	**TsAP-S1** FLSLIP**K**LV**KK**II**K**AFK-NH_2_	Ser, Ile, and Gly by Lys	0.66^b^	0.75	+6	Guo et al. (2013)
	**TsAP-2** FLGMIPGLIGGLISAFK- NH_2_	0.90^b^	0.51	+2	**TsAP-S2** FLGMIP **K** LI **KK** LI **K** AFK-NH_2_	Ser and Gly by Lys	0.67^b^	0.73	+6	
*Heterometrus petersii*	**Hp1404** GILGKLWEGVKSIF-NH_2_	0.68^b^	0.67	+1	**Hp1404-T1** ILGKLWEGVKSI-NH_2_	Removal of Gly and Phe	0.65^b^	0.69	+1	Kim et al. (2018)
					**Hp1404-T1a** IL **K** KLWEGVKSI-NH_2_	Gly by Lys	0.58^b^	0.76	+2	
					**Hp1404-T1b** ILKKL **L** EGVKSI-NH_2_	Trp by Leu	0.52^b^	0.75	+2	
					**Hp1404-T1c** ILKKLL **K** GVKSI-NH_2_	Glu by Lys	0.49^b^	0.78	+4	
					**Hp1404-T1d** ILKKLLK **K** VKSI-NH_2_	Gly by Lys	0.41^b^	0.76	+5	
					**Hp1404-T1e** ILKKLLKKVK **K** I-NH_2_	Ser by Lys	0.33^b^	0.83	+6	
*Androctonus crassicauda*	**AcrAP1** FLFSLIPHAISGLISAFK	0.90	0.43	+2	**AcrAP1a** FLF **K** LIP **K** AI **K** GLI **K** AFK	Ser and His by Lys	0.68	0.64	+6	Du et al. (2014)
	**AcrAP2** FLFSLIPNAISGLLSAFK	0.85	0.47	+2	**AcrAP2a** FLF **K** LIP **K** AI **K** GLL **K** AFK	Ser and Asn by Lys	0.67	0.63	+6	
*Pandinus imperator*	**Pin2** FWGALAKGALKLIPSLFSSFSKKD	0.54^b^	0.48	+3	**Pin2 [G]** FWGALAKGALKLI **G** SLFSSFSKKD	Pro by Gly	0.51^b^	0.49	+3	Rodríguez et al. (2014)
					**Pin2 [GPG]** FWGALAKGALKLI **GPG** SLFSSFSKKD	Addition and substitution of Pro, Ser, and Leu by Gly and Pro	0.49^b^	0.28	+3	
*Androctonus aeneas*	**AaeAP1** FLFSLIPSVIAGLVSAIRN	0.84	0.45	+2	**AaeAP1a** FLF **K** LIP **KA** I **K** GLV **K** AIR **K**	Ser, Ala, and Asn by Ala and Lys	0.61	0.66	+7	Du et al. (2015)
	**AaeAP2** FLFSLIPSAIAGLVSAIRN	0.80	0.42	+2	**AaeAP2a** FLF **K** LIP **KV** I **K** GLV **K** AIR **K**	Ser, Ala, and Asn by Val and Lys	0.56	0.64	+7	
*Didymocentrus krausi*	**MK049518** FLGLLGSVLGSVLPSIFK-NH_2_	0.88^b^	0.46^b^	+1	**S3K** FLGLLG **K** VLG **K** VLP **K** IFK-NH_2_	Ser by Lys	0.72^b^	0.57^b^	+4	Li et al. (2020)
					**G2K–S3K** FL **K** LL **KK** VL **KK** VLP **K** IFK-NH_2_	Ser and Gly by Lys	0.56^b^	0.62^b^	+7	
*Vejovis mexicanus* and *Hadrurus gertschi*	**Vejovine** GIWSSIKNLASKAWNSDIGQSLRNKAAGAINKFVADKIGVTPSQAAS and **Hadrurin** GILDTIKSIASKVWNSKTVQDLKRKGINWVANKLGVSPQAA	0,29^b^ and 0,32^b^	0,081^b^ and 0,13^b^	+4 and +5	**Δ(A29)** GILKTIKSIASKVANTVQ **KLKRK** AKNAV	Sequence combination	0,17	0,50	+8	Sánchez-Vásquez et al. (2013)
					**Δ (K12-Q18; N26-A29)** GILKTIKSIAS **KLKRK** AK	Sequence combination	0,27	0,63	+6	
					**K4N Δ (K12-Q18; N26-A29)** GILNTIKSIAS **KLKRK** AK	Sequence combination	0,29	0,61	+5	

H, hydrophobicity; µH, hydrophobicity moment; Z, net charge.

a Modifications in analog peptides are marked in bold.

b Not calculated by the authors of the studies (calculated here using the HeliQuest server).

**TABLE 2:** Comparison of hemolytic and antimicrobial activities of native scorpion peptides and their analogs after modifications.

Native peptide	Hemolytic activity^a^	MIC	Analog peptide	Hemolytic activity^a^ (∼30%)	MIC	Strains	References
Stigmurin	1.17% in 75 µM	>150; 9.3 and 37.5 µM	StigA6	30% in 75 µM	4.6; 2.3 and 9.3 µM	*E. coli*, *S. aureus*, and *C. albicans*, respectively	Parente et al. (2018)
			StigA16	30% in 75 µM	2.3; 2.3 and 4.6 µM		
			StigA25	30% in 18.8 µM	2.3; 1.2 and 9.4 µM	*E. coli*, *S. aureus*, and *C. albicans*, respectively	Amorim-Carmo et al. (2019)
			StigA31	30% in 18.8 µM	1.2; 2.3 and 4.7 µM		
VmCT1	Not calculated for 30%	0.78; 3.12 and 12.5 µM	[K]3-VmCT1-NH2	Not calculated for 30%	0.39; 3.12 and 3.12 µM	*P. aeruginosa*, *S. aureus*, and *C. albicans*, respectively	Pedron et al. (2017)
			[K]7-VmCT1-NH2	Not calculated for 30%	0.39; 1.56 and 1.56 µM		
			[K]11-VmCT1-NH2	Not calculated for 30%	0.39; 1.56 and 1.56 µM		
			[E]4-VmCT1-NH2	Not calculated for 30%	3.12; 50 and 50 µM		
			[E]7-VmCT1-NH2	Not calculated for 30%M	0.78; 6.25 and 12.5 µM		
			[W]9-VmCT1-NH2	Not calculated for 30%	0.78; 1.56 and 1.56 µM		
			[E]4[W]9-VmCT1-NH2	1.6 µM	3.12; 12.5 and 6.25 µM		
AamAP1	∼30% in 100 µM	150; 20 and 64 µM	AamAP-S1	∼30% in 40 µM	5; 3 and 5 µM	*E. coli*, *S. aureus*, and *C. albicans*, respectively	Almaaytah et al. (2012)
		20 µM	A3	∼30% in 40 µM	5 µM	*S. aureus*	Almaaytah et al. (2018)
		20 and 150 µM	AamAP1-Lysine	∼30% in 80 µM	5 and 7.5 µM	*S. aureus* and *E. coli*, respectively	Almaaytah et al. (2014)
IsCT1	30% in ∼20 μg/ml	50; >100 and 50 μg/ml	IsCT1A1	∼5% in 100 μg/ml	>100; >100 and >100 μg/ml	*S. aureus*, *B. cereus*, and *E. coli*, respectively	de la Salud Bea et al. (2017)
			IsCT1V1	∼5% in 100 μg/ml	>100; >100 and >100 μg/ml		
			IsCT1L1	30% in ∼20 μg/ml	50; >100 and 50		
			IsCT1K7	30% in ∼30 μg/ml	100; >100 and >100 μg/ml		
			IsCT1E7	0% in 100 μg/ml	>100; >100 and >100 μg/ml		
IsCT2	30% in ∼20 μg/ml	50; 100 and 50 μg/ml	IsCT2A1	∼5% in 100 μg/ml	>100; >100 and >100 μg/ml		
			IsCT2V1	∼20% in 100 μg/ml	>100; >100 and >100 μg/ml		
TsAP-1	∼5% in 160 µM	120; 160 and 160 µM	TsAP-S1	30% in ∼6 µM	2.5; 5 and 2.5 µM	*S. aureus*, *E. coli*, and *C. albicans*, respectively	Guo et al. (2013)
TsAP-2	30% in ∼30 µM	5; >320 and 10 µM	TsAP-S2	30% in ∼6 µM	5; 5 and 2.5 µM		
Hp1404	30% in ∼40 µM	12.5 µM	Hp1404-T1	N/C	>25 µM	*P. aeruginosa* ATCC 27853	Kim et al. (2018)
			Hp1404-T1a	N/C	>25 µM		
			Hp1404-T1b	N/C	>25 µM		
			Hp1404-T1c	N/C	3.13 µM		
			Hp1404-T1d	N/C	1.56 µM		
			Hp1404-T1e	0% in 200 µM	1.56 µM		
AcrAP1	Not calculated for 30%	8; >250 and 16 µM	AcrAP1a	Not calculated for 30%	4; 8 and 4 µM	*S. aureus*, *E. coli*, and *C. albicans*, respectively	Du et al. (2014)
AcrAP2	Not calculated for 30%	8; >250 and 16 µM	AcrAP2a	Not calculated for 30%	4; 8 and 4 µM		
Pin2	30% in ∼5 µM	18.8 and 37.5 µM	Pin2 [G]	30% in ∼2 µM	12.5 and 12.5 µM	*E. coli* and *S. aureus*, respectively	Rodríguez et al. (2014)
			Pin2 [GPG]	30% in ∼25 µM	25 and 25 µM		
AaeAP1	Not calculated for 30%	16; >512 and 32 mg/L	AaeAP1a	Not calculated for 30%	4; 16 and 4 mg/L	*S. aureus*, *E. coli*, and *C. albicans*, respectively	Du et al. (2015)
AaeAP2	Not calculated for 30%	16; >512 and 32 mg/L	AaeAP2a	Not calculated for 30%	4; 1 and 4 mg/L		
MK049518	Not calculated	6.7; >54.1 and >54.1 µM	S3K	Not calculated	1.5; 12.6 and 12.6 µM	*S. aureus*, *E. coli*, and *P. aeruginosa*, respectively	Li et al. (2020)
			G2K–S3K	Not calculated	1.4; 2.8 and 5.7 µM		

a 30% of hemolysis was considered the maximum acceptable limit.

### Hydrophobic Moment

Membrane-active peptides generally present two characteristics: they must be soluble in water to enable transport and to present hydrophobic residues to interact with the cytoplasmic membrane (Dathe and Wieprecht, 1999). Different from hydrophobicity, the hydrophobic moment (µH) is the hydrophobicity of a peptide measured for different angles of rotation per residue; in other words, it is the quantification of the peptide amphipathicity, where a face of the peptide helix is hydrophilic and the other is hydrophobic (Segrest et al., 1990; Cherry et al., 2014).

AMPs usually present high hydrophobic moment and moderate hydrophobicity (Wallace et al., 2004). A more direct correlation between the hydrophobic moment and the antimicrobial activity is observed, so that when the hydrophobic moment increases, the antimicrobial activity increases (Lee et al., 2004; Almaaytah et al., 2012; la Salud Bea et al., 2015). One example is the study by la Salud Bea and contributors (2015) in which they designed 5 analog peptides from the AMP BmKn1 from the scorpion *Buthus martensii*, where the analog peptides with higher hydrophobic moments revealed increased antimicrobial activity. Similar results were found by Parente and collaborators (2018), where two peptide analogs studied with hydrophobic moments of 0.66 and 0.72 showed higher antimicrobial activity and a broader activity spectrum than the native peptide, which showed a hydrophobic moment of 0.57 (Parente et al., 2018).

Along these lines, we can observe that the hydrophobic moment is a feature that modulates AMPs’ activity through hydrophobic interactions between the peptide and bacterial membrane.

### Net Charge

Despite the diverse nature of AMPs, the cationic character is a feature broadly found among them (Epand and Epand, 2011). The net charge is the sum of the charges from all ionizable groups of a peptide (Bahar and Ren, 2013). Gram-positive or Gram-negative bacterial membrane is negatively charged; therefore, the main role of the presence of cationic amino acids in AMPs is to interact with these membranes (Andersson et al., 2016). Electrostatic interactions between cationic peptides and anionic phospholipids present in the bacterial membrane play a major role in the recognition and selectivity of microbial surface (Malanovic and Lohner, 2016).

**FIGURE 3:**
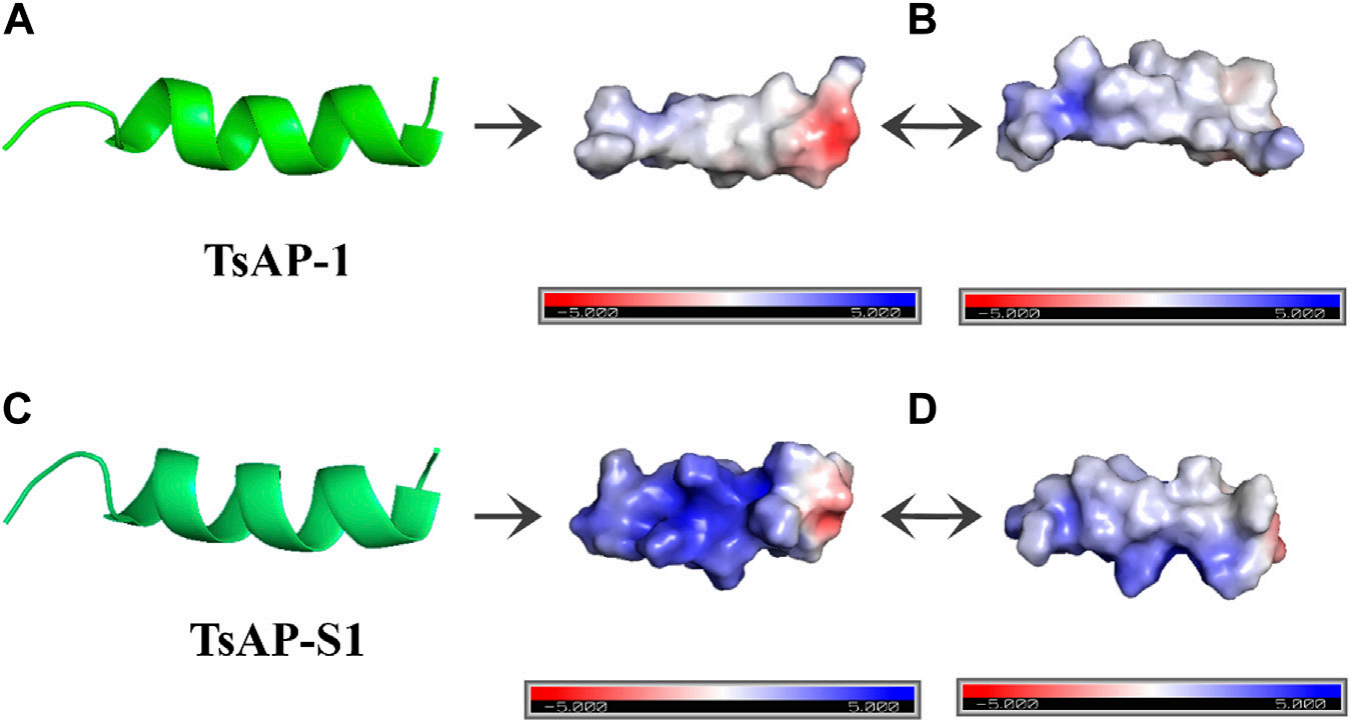
Antimicrobial peptides visualized using PyMol. **(A,C)** Peptide tridimensional view of TsAP-1 and TsAP-S1, respectively. **(B,D)** Different angles of the electrostatic surface created through Adaptive Poisson–Boltzmann solver (APBS). The negatively charged surface is shown in red, the positively charged surface is shown in blue, and the neutrally charged surface is shown in white.

The net charge of AMPs can vary substantially from 0 to +16. Although the net charge of most AMPs ranges within positive intermediate values, there are also reports of negatively charged AMPs. However, the net charge of most AMPs ranges from +4 to +6, with an average of +2.26 for linear peptides, which may represent an optimal range for their activity (Huang et al., 2010; Wang et al., 2016). There are exceptions for this range, as the AMPs imcroporin and pandinins from *Isometrus maculatus* and *P. imperator*, respectively, both present a net charge of +1 (Zhao et al., 2009; Zeng et al., 2013). It is known that the bacterial membrane is negatively charged and it is approximately 50% more negative than mammalian cell membranes (Benarroch and Asally, 2020). The charge difference in prokaryotic and eukaryotic cell membranes increases the selectivity of the cationic AMPs (Jenssen et al., 2006). To illustrate changes in charge distribution on the surface of AMPs, the peptide TsAP-1 from *T. serrulatus* with charge +2 and its analog TsAP-S1 with charge +6 were analyzed (Figure 3) (Guo et al., 2013).

The charge is directly related to the antimicrobial activity and regulates the balance between antimicrobial and hemolytic activities. A study observed that the antimicrobial activity was affected in the analogs of the AMP IsCT designed to be more negatively charged; however, cytotoxicity was not observed in the tested concentrations (de la Salud Bea et al., 2017). Moreover, anionic or acidic AMPs from scorpions are less commonly studied; however, they are present in all detected species, for instance, TanP from *T. stigmurus* and Hap-1 (Shi et al., 2018; Melo et al., 2021).

### Polar Angle (*θ*_*p*_) and Hydrophobic Angle (*θ*_*h*_)

Polar (*θ*_*p*_) and hydrophobic (*θ*_*h*_) angles are defined by the polar and hydrophobic faces of the amphipathic peptides, and they modulate the way these peptides associate with lipidic membranes. Thus, they are an important parameter for understanding peptide–membrane interactions, as they emphasize the difference between bonding and permeabilization. Peptides with low polar (or hydrophilic) angles and high hydrophobic means tend to form transmembrane pores. Meanwhile, peptides with equivalent polar and nonpolar faces (amphipathic) tend to bind parallelly to the membrane, with the hydrophobic face interacting with lipids (Dathe and Wieprecht, 1999). Regardless of the hydrophobicity or the hydrophobic moment, it is expected that the hydrophobic/hydrophilic angle will influence the peptide’s insertion in the membrane and the transmembrane pore structures (Uggerhøj et al., 2015).

A study showed that leucine-rich AMPs can insert deeper in the lipid bilayer. Conversely, peptides with lower leucine content were only capable of interacting with the surface of negatively charged membranes. Thus, a broad hydrophobic core is a prerequisite to an effective disturbance of the cellular membrane (Kiyota et al., 1996).

To study how the polar angle affects AMPs, 3 peptides were modified to have three different polar angles each: 100°, 120°, and 140°. The authors observed that decreased hemolytic activity was present when the peptides had higher polar angles (Tytler et al., 1993). This effect was also observed for analogs of the scorpion peptide VmCT1, where the increase in the polar angle by the substitution of alanine by glutamic acid residues at the hydrophilic face was important to reduce hemolytic activity by approximately 15 times for the analog [E]^4^-VmCT1-NH_2_ (Pedron et al., 2017).

It was also observed that the balance between the hydrophobic and hydrophilic faces in the analogs of the scorpion peptide Pin2 induced a lower hemolytic activity (Rodríguez et al., 2014), making it evident that alterations in the balance of the polar and nonpolar faces mainly affect the membrane permeabilization efficiency and the reduced amphipathicity can, at least partially, decrease hemolytic activity. Uggerhøj and collaborators (2015) concluded that the general activity of an α-helical AMP is modulated by the strength of the interaction of its hydrophilic face with the lipid head groups and the solvent, and by the strength of the interaction of its hydrophobic face with the interior of the membrane.

## General Antimicrobial Peptide Molecular Targets

The main mechanism of action of AMPs is associated with their capacity to cause membrane damage. The interaction between AMPs and microorganisms occurs due to electrostatic forces between their positive amino acid residues and the negative charges exposed at the pathogen surfaces (Almaaytah and Albalas, 2014). However, most studies neglect the fact that microbial cells have cell walls, component that can be a cellular target or contain molecular targets for AMPs, besides being a defensive physical barrier against antibiotic molecules (Peschel and Sahl, 2006).

Cell wall is the bacterial structure responsible for the shape of bacterial cells, preventing lysis due to high cytoplasmic osmotic pressure. Moreover, it permits anchoring of membrane components and extracellular proteins. The major component of the Gram-positive bacteria cell wall is the peptidoglycan associated with teichoic (TA) and lipoteichoic acid (LTA), which are responsible for the negative net charge in these bacteria (Scott et al., 1999). Differently, Gram-negative bacteria present an outer membrane, composed mainly of negatively charged lipopolysaccharides (LPS), overlapping the peptidoglycan. The set of LPS provides a protection barrier against the insertion of molecules with molecular weight higher than 1,000 Da. It also provides a defense mechanism against antibiotics and AMPs as larger molecules cannot translocate through the membrane (Kotra et al., 1999).

It is possible that the cationic and amphiphilic nature of AMPs allows these peptides to accumulate on the negatively charged bacterial surface. In such a way, AMPs can promote cell wall destabilization by interacting with microbial surface components (Brogden, 2005). This interaction mechanism between AMPs and pathogen cell wall components has already been described for various peptides. For example, Kn2-7, an analog peptide from BmKn2, a native peptide from the scorpion *M. martensii*, promoted the disruption of *Staphylococcus aureus* and *Escherichia coli* cell walls through interaction with LTA and LPS, respectively (Cao et al., 2012). In another study, it was observed that the analogs of Bac2A generated drastic changes at the surface of *S. aureus* cells, such as protuberances and fissures at the cell wall, but interferences on the cellular membrane were not observed. Bac2A analogs only had effect on the cell wall, inhibiting *S. aureus* without cellular lysis (Wu X. et al., 2014).

**FIGURE 4:**
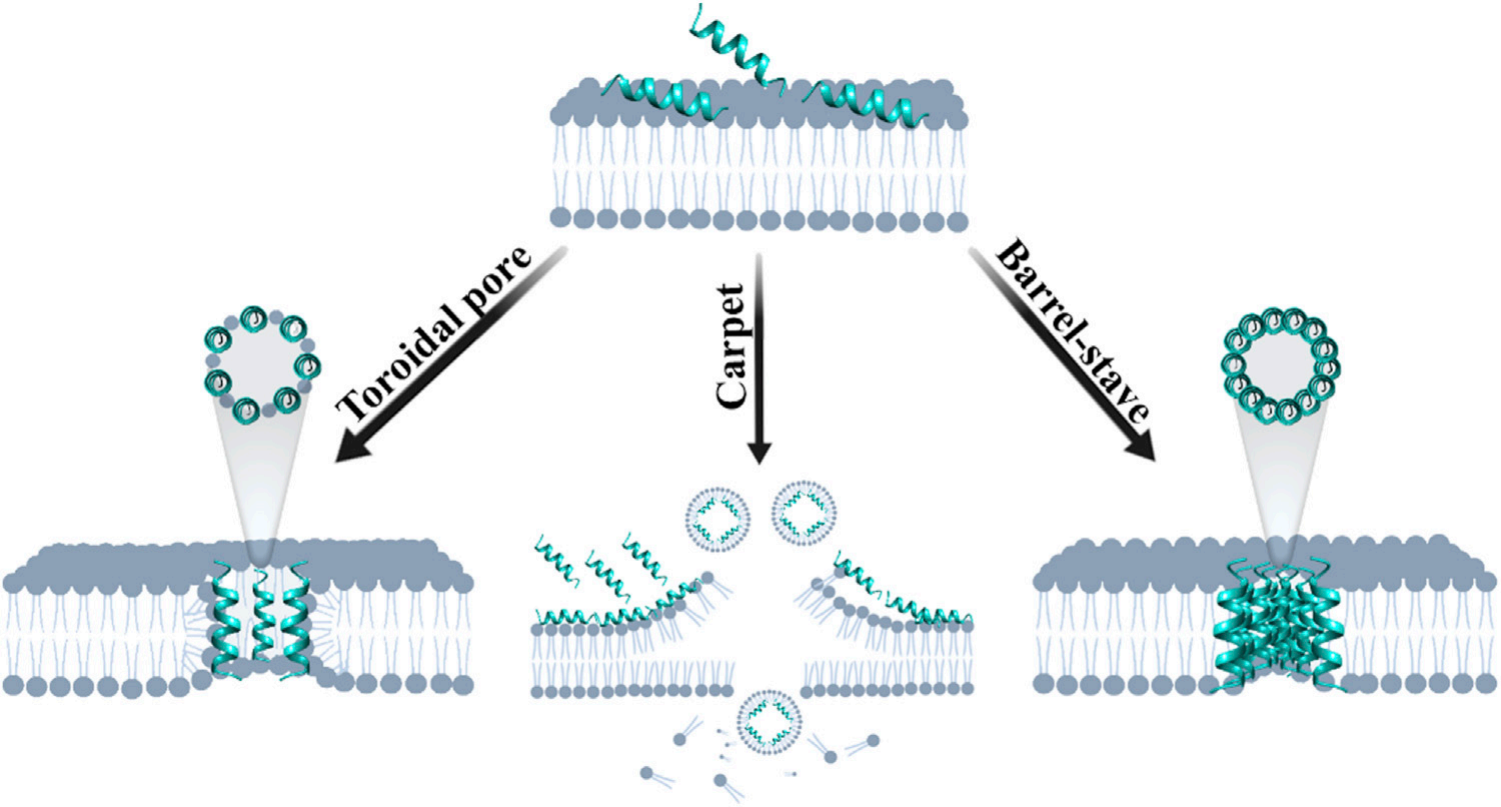
Schematic models of the main proposed mechanisms of action of AMPs on microorganism cellular membranes.

Mainly negatively charged lipids, such as phosphatidylglycerol (PG), cardiolipin, and the zwitterionic phosphatidylethanolamine (PE), compose the bacterial cellular membrane. The interaction and action of these peptides against target cells depend not only on the cell surface but also on the AMP’s amino acid composition. This hypothesis is supported by the conserved positive amino acid residues in AMP sequences from several organisms (Guilhelmelli et al., 2013). Moreover, the peptide’s secondary structure is essential for the interaction with anionic phospholipids (Matsuzaki, 2009). AMPs can induce various membrane defects, and the most characterized are pore formation, phase separation, and lipid bilayer rupture (Seyfi et al., 2020). Some models have been proposed to explain the membrane rupture caused by AMPs (Figure 4).

In the toroidal model, the peptides insert in the membrane forming a beam, inducing the lipids to bend continuously across the pore. As a consequence, membrane lipids become interleaved with the peptides participating in the pore’s structure (Yang et al., 2001; Yeaman and Yount, 2003). The carpet model is characterized by the overlay of AMPs at the membrane surface, acting as an emulsifier affecting the membrane’s structure (Rotem and Mor, 2009). This interaction is initiated by electrostatic attraction, and then the density of AMPs on the membrane surface increases until a threshold is reached, leading to membrane disintegration, causing cell lysis and micelle formation (Oren and Shai, 1998; Seyfi et al., 2020). The Barrel–Stave model states that AMP monomers interact with the cell surface, where they oligomerize creating a pore. The recruitment of additional monomers can enlarge the pore, promoting cellular extravasation and cell death. In this mechanism, the peptide’s secondary structures, such as α-helix and amphipathic β-sheet, are essential for pore formation (Breukink and de Kruijff, 1999). Hydrophobic peptide regions interact with the membrane lipids, while the hydrophilic regions interact forming the pore lumen. One of the major differences between the toroidal pore and the Barrel-Stave pore is the participation of phospholipids forming the pore along with AMPs in the former, whereas the latter is formed only by peptides (Brogden, 2005; Nguyen et al., 2011).

Furthermore, some AMPs can target intracellular components such as nucleic acid and proteins. It has been reported that AMPs Smp24 and Smp43 from the Egyptian scorpion *Scorpio maurus palmatus* not only disrupt bacterial cell membranes but can also interfere with intracellular gene expression (Tawfik et al., 2021). A study using radioactive isotopes clearly showed that I9K-IsCT and E7K, which are analogs of IsCT from the scorpion *O. madagascariensis*, inhibit nucleic acids and protein synthesis in *E. coli* (Tripathi et al., 2015). This strengthens the idea that a single AMP can show multiple mechanisms of action.

**FIGURE 5:**
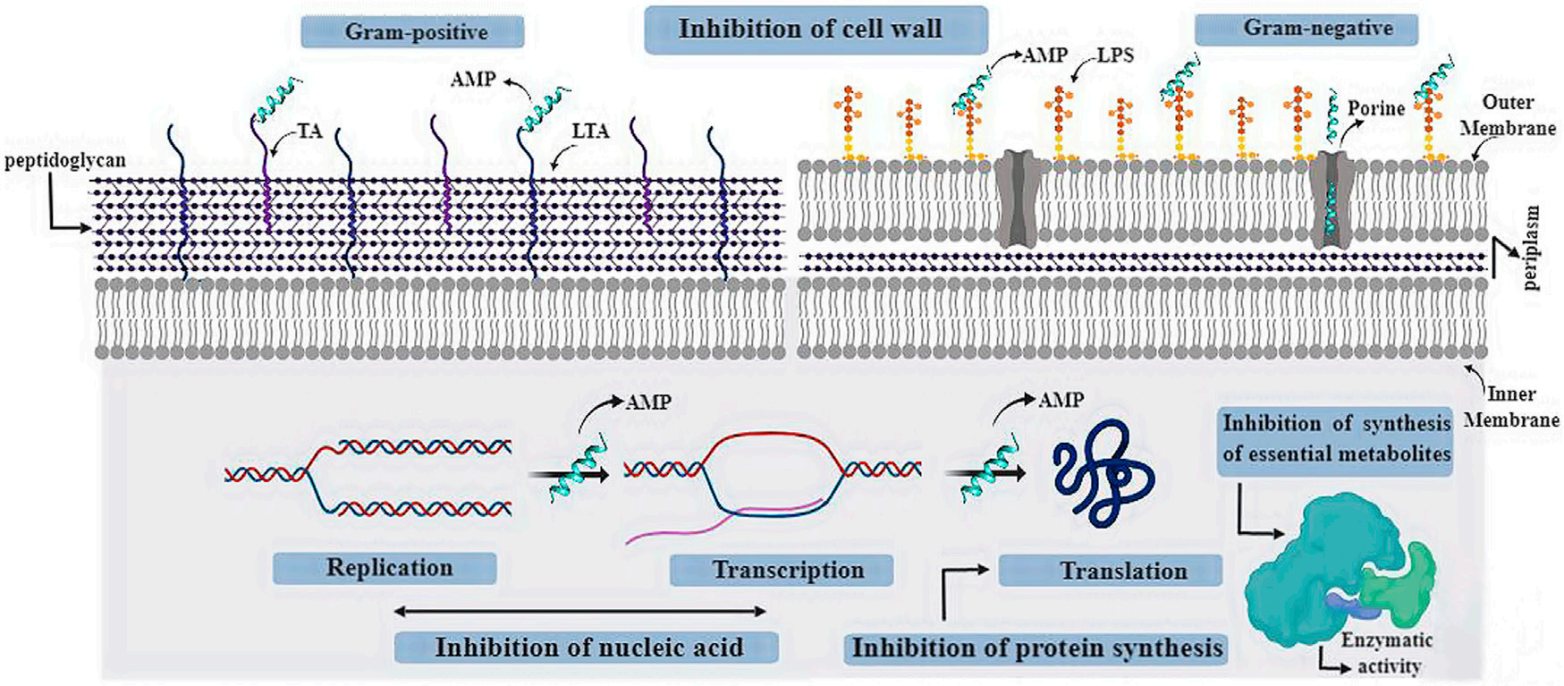
Illustration of the main molecular targets of AMPs already described. AMP, antimicrobial peptide; TA, teichoic acid; LTA, lipoteichoic acid.

Nevertheless, some peptides are too small and cannot lead to pore formation, but instead, they can cause membrane disturbance, such as thinning, and modified electrostatics. These peptides can spread to the cytoplasm and bind to intracellular targets, altering natural events, such as cell wall synthesis and cell division. These molecular interactions occur due to the chemical composition of the molecule, in addition to structural factors such as size and secondary structure (Fjell et al., 2012) (Figure 5).

## Structural Analysis Through Nuclear Magnetic Resonance

Nuclear magnetic resonance spectroscopy is a technique used to study the atomic structure of different molecules. Thus, analyzing AMP structures through NMR spectroscopy can help to infer structures and shed light on their mechanism of action at the atomic level (Nomura et al., 2014).

Liquid-state NMR spectroscopy is widely used to study the structure of these peptides (Lee et al., 2004; Gao et al., 2009; Daniele-Silva et al., 2021). To that end, solvents such as water, TFE, and SDS can be used to better understand their solubility and flexibility structure in different environments. But solid-state NMR is an approach used to analyze their interaction with membrane mimetics, such as vesicles containing different phospholipids, with phosphatidylcholine or phosphatidylglycerol being the most used. By altering the proportion of these lipids and using solid NMR analysis, it is possible to assess their interaction with different vesicle compositions, and thus, the specificity of these peptides (Bandyopadhyay et al., 2014; Nomura et al., 2014).

Table 3 shows a series of AMPs from scorpion venoms that have been analyzed by NMR spectroscopy, the equipment information, and solvent used, as well as the overall structure result. It is possible to note from this table that AMPs from scorpions are mostly studied and analyzed using liquid-state NMR spectrometer functioning at 500–800 MHz, in water or TFE solution or in SDS or DPC micelles. When analyzing the same peptide in different solvents, it is possible to notice changes in the percentage of structure obtained; for example, ctriporin showed 73.6% of α-helix in SDS and 52.6% of α-helix in DPC micelles (Bandyopadhyay et al., 2014). Therefore, it is important to assess the structure of these peptides in an environment that better mimics membranes to acquire the more accurate structure they show when acting on microbial membranes.

**TABLE 3:** Comparison of the structure of antimicrobial peptides from scorpion venom analyzed by nuclear magnetic resonance spectroscopy.

Peptide/references	Sequence^a^	Equipment	Solvent	Structure
**Androctonin**/Mandard et al. (1999)	RSVCRQIKICRRRGGCYYKCTNRPY	Liquid probe—500 MHz	H_2_O:D_2_O (9:1)	β-Hairpin
**Meucin-13**/Gao et al. (2009)	IFGAIAGLLKNIF	Liquid probe—600 MHz	H_2_O:d_3_-TFE (1:1)	61.5% α-helix
**Meucin-18**/Gao et al. (2009)	FFGHLFKLATKIIPSLFQ	Liquid probe—600 MHz	H_2_O:d_3_-TFE (1:1)	76.4% α-helix
**Meucin-24**/Gao et al. (2010)	GRGREFMSNLKEKLSGVKEKMKNS	Liquid probe—600 MHz	H_2_O:d_3_-TFE (1:1)	70.8% α-helix
**Stigmurin**/Daniele-Silva et al. (2021)	FFSLIPSLVGGLISAFK	Liquid probe—800 MHz	H_2_O:d_3_-TFE (3:2)	58.8% α-helix
**IsCT**/Lee et al. (2004)	ILGKIWEGIKSLF	Liquid probe—600 MHz	D_25_-SDS	46.1% α-helix
**[A** ^**6**^ **]-IsCT**/Lee et al. (2004)	ILGK **A** WEGIKSLF	Liquid probe—600 MHz	D_25_-SDS	Random structure
**[K** ^**7**^ **]-IsCT**/Lee et al. (2004)	ILGKIW **K** GIKSLF	Liquid probe—600 MHz	D_25_-SDS	84.6% α-helix
**[K** ^**7**^ **, P** ^**8**^ **, K** ^**11**^ **]-IsCT**/Lee et al. (2004)	ILGKIW **K** IK **K** LF	Liquid probe—600 MHz	D_25_-SDS	46.1% α-helix
**Pandinin 2**/Corzo et al. (2001)	FWGALAKGALKLIPSLFSSFSKKD	Liquid probe—500 and 600 MHz	DPC micelles	79.1% α-helix
**Ctriporin**/Bandyopadhyay et al. (2014)	FLWGLIPGAISAVTSLIKK	Liquid probe—800 MHz	SDS	73.6 α-helix
**Ctriporin**/Bandyopadhyay et al. (2014)	FLWGLIPGAISAVTSLIKK	Cryoprobe—800 MHz	DPC micelles	52.6 α-helix
**Pin1**/Nomura et al. (2014)	GKVWDWIKSAAKKIWSSEPVSQLKGQVLNAAKNYVAEKIGATPT	Solid probe—300 MHZ	PC or PC/PG vesicles	2 α-helix separated by 8 aa in random structure

a Modifications in analog peptides are marked in bold.

Exploring the structure of AMPs using the NMR technique can also improve the rational design of new molecules, as it makes it possible to observe the amino acid residues important to the structure assembly and the antimicrobial activity. When Lee and collaborators (2004) studied the peptide IsCT and its analogs, they observed that the substitution of tryptophan by alanine at position 6 of the sequence resulted in a change from an α-helix to an unstructured peptide and a dramatic decrease in the antimicrobial activity, highlighting the importance of this residue for the structure-activity of this peptide. On the other hand, another analog of the IsCT peptide in which glutamic acid was substituted by lysine showed a longer α-helix and higher antimicrobial activity, proving it to be a satisfactory modification.

Solid-state NMR is a technique that can provide results for a better understanding of the mechanism of action of AMPs. The interaction of Pin1 with vesicles containing phosphatidylcholine (PC) was analyzed, and the results demonstrated the formation of a cubic phase. The cationic portion of a choline headgroup from the vesicles binds with the aromatic sidechain of the tryptophan, whereas when studying an analog in which tryptophan was substituted by an alanine residue, the cubic phase was not observed, indicating the importance of tryptophan for the Pin1 mechanism of action (Nomura et al., 2014). Through the solid-state NMR analysis of this peptide, Nomura and collaborators (2014) were also able to evaluate the specificity of Pin1 by analyzing its interaction with vesicles containing only phosphatidylcholine and containing both phosphatidylcholine and phosphatidylglycerol; in the latter, Pin1 did not induce the cubic phase, showing interaction with only PC vesicles as it did in vesicles containing only PC. These studies convey the significance of NMR spectroscopy studies of AMPs as an analytical tool to assess their structure and mechanism of action.

## Amino Acid Substitutions and Their Effect on Antimicrobial and Hemolytic Activities

### Amino Acid Classification Based on Side Chain (R) Polarity

Amino acids can be classified based on the polarity of their side chain. According to that, amino acids can be nonpolar (hydrophobic or insoluble in water), neutral polar (hydrophilic or soluble in water), negatively charged (acidic), and positively charged polar (basic) (Lee et al., 2011). Polarity is the capacity of a chemical group to form electrical poles, which enables solubilization in water by interacting with water molecules by the formation of hydrogen bonds. Nonpolar amino acids are insoluble in water due to their hydrocarbon organic chains, which are unable to form hydrogen bonds with water molecules. Polar amino acids are soluble in water because of their polar side groups, such as OH, NH, and C = O. Basic amino acids present a positive net charge at pH 7.0, while acidic amino acids show a negative net charge at pH 7.0. This information allows several rational substitutions in the peptide’s primary structure according to the properties one aims to change (Barth, 2000; Moreira et al., 2007). Modifications for scorpion peptides are listed in Table 1.

### Basic Amino Acid Residue Modification

Replacement of amino acids by basic amino acid residues in AMPs, such as lysine and arginine, confers a higher positive charge, as they are positively net charged in neutral pH. The positive side chains of amino acids are externally oriented on α-helical peptides, as they make up the helice’s hydrophilic face (Hancock, 1997; Amorim-Carmo et al., 2019).

Numerous approaches have been performed to evaluate the positive charge influence on antimicrobial activity (Table 1), and it is observed that increased antimicrobial activity correlates with increased cationicity (Table 2). The cationicity increase is usually associated with higher antibacterial activity (la Salud Bea et al., 2015).

Stigmurin, a peptide from the scorpion *T. stigmurus*, was modified by substituting serine and glycine to lysine residues. These replacements resulted in an increase in the net charge from +2 to +7, and also higher antimicrobial activity and broadened activity spectrum (Parente et al., 2018; Amorim-Carmo et al., 2019). Stigmurin did not show activity against Gram-negative bacteria, but it was observed that with the increased positive charge generated by lysine substitution, its analogs demonstrated potent antimicrobial activity against several Gram-negative strains with an MIC of 2.4 µM (Parente et al., 2018).

A similar replacement was performed in the AMP from the scorpion *Androctonus amoreuxi* venom denominated AamAP1. By replacing histidine with lysine in the native peptide AamAP1, the analog named AamAP-S1 was produced. This substitution resulted in a higher net charge, while other structural parameters related to its activity were not altered. AamAP-S1 showed a remarkable increase in antimicrobial activity and activity spectrum. To illustrate that, the sensibility of *E. coli* to AamAP-S1 was 25-fold higher than to AamAP1. A similar increase in activity was observed when the analog was tested against the Gram-positive bacteria *S. aureus* and the yeast *Candida albicans* (Almaaytah et al., 2012).

To analyze how changes in net charge affect antimicrobial activity, analogs of the peptide VmCT1 from the scorpion *Vaejovis mexicanus smithi* were generated by substituting glycine, asparagine, and serine with lysine at the hydrophilic face. These analogs also had higher antimicrobial activity when compared with the native peptide. Substitution of asparagine by lysine in the [K]^7^-VmCT1-NH_2_ analog showed an MIC value between 0.39 and 12.5 µM against *B. subtilis* and *C. albicans*; this represents an 8-fold increase when compared to the native peptide VmCT1. These substitutions reassert that cationicity is one of the most important physicochemical parameters related to strong interactions between AMPs and microorganisms, which is crucial to the antimicrobial activity (Pedron et al., 2017).

### Hydrophobic Residue Modifications

The R group of nonpolar amino acids is hydrophobic. It is known that peptides have a polycationic and hydrophobic nature and that these properties are important to the initial interaction between AMPs and bacterial cell membrane (Hancock et al., 1995; Wu et al., 1999; Andrade and Colherinhas, 2020).

The amino acid leucine can enhance hydrophobicity in an amphipathic structure (i.e., α-helices), playing an important role in antimicrobial activity through the interaction with bacterial membranes (Kim et al., 2018). Based on the nature of the AMP, it is expected that by increasing the hydrophobicity of the peptide, the helicity will increase, resulting in greater antimicrobial activity; however, increased hemolytic activity can also be observed (Yeaman and Yount, 2003; Parente et al., 2018; Amorim-Carmo et al., 2019).

A study evaluated the influence of the hydrophobicity enhancement on scorpion AMPs, and it was observed that, in general, higher hydrophobicity corresponds to the increase in antimicrobial and hemolytic activities (de la Salud Bea et al., 2015). Another study observed that the substitution of hydrophobic amino acids to the AMP BmKn2 results in an increased helicity and higher antimicrobial activity. These improvements in activity were attributed to higher hydrophobicity in the analogs (de la Salud Bea et al., 2017).

Increased antimicrobial activity was also observed for the analog from IsCTL1 from *O. madagascariensis* with leucine substitutions. These analogs inhibited *S. aureus* growth at concentrations around 20–25 μg/ml, while *E. coli* inhibition occurred at concentrations of 15–25 µg/ml. On the other hand, the analog with valine substitutions inhibited bacterial growth at concentrations lower than 10 µg/ml (de la Salud Bea et al., 2017). In general, the results showed that increased hydrophobicity, as a result of alanine, valine, and leucine substitutions, enhanced antibacterial activity. However, it also incremented the peptide’s hemolytic activity (de la Salud Bea et al., 2017).

### Polar (Uncharged) Residue Modifications

Polar uncharged residue R groups are soluble in water because of their functional groups that bind to water molecules. The main amino acids classified as polar are serine, threonine, cysteine, asparagine, and glutamine. Glycine has the simplest structure, and although it is easily classified as a nonpolar amino acid, its side chain is too small, so it does not really contribute to hydrophobic interactions (Nelson et al., 2008). Frequently, uncharged amino acids are substituted by basic or hydrophobic residues when one intends to increase charge and hydrophobicity, respectively. For scorpion analog peptides, this type of substitution is usually observed (Table 1). Substitution of neutral residues (serine and asparagine for lysine) on the hydrophilic face resulted in more effective MIC values when compared to the native peptide: the analog [K]^7^-VmCT1-NH_2_ presented an MIC value varying from 0.39 to 12.5 µM, and [K]^11^-VmCT1-NH_2_ showed an MIC between 0.39 and 6.25 µM. But the most significant antimicrobial activity was obtained against *P. aeruginosa* (0.78, 0.39, and 0.39 µM, respectively) (Pedron et al., 2017).

By substituting serine residues for cationic residues, Du and contributors (2014) observed enhanced antifungal activity (MICs from 16 to 4 µM) for the analog peptides of AcrAP1 and AcrAP2 from the scorpion *Androctonus crassicauda*. The authors tested the activity against Gram-positive bacteria and yeasts and observed increased activity spectrum for Gram-negative bacteria (MIC 8 µM), as the opposite of the native peptides, which did not show activity against Gram-negative strains. Enhanced activity spectrum was also demonstrated by Parente and contributors (2018) using Stigmurin as the prototype, a higher activity spectrum for Gram-negative bacteria was achieved by the substituting neutral for basic residues.

A study evaluated the influence of glycine at the Pandinin 2 (Pin2) sequence, which is a highly hemolytic peptide with a central proline residue. This amino acid forms a kink at the center of the structure that is related to its pore-forming activity in human erythrocytes (Rodríguez et al., 2014). To decrease hemolytic activity, the authors replaced the Pro14 residues in Pin2 with glycine residues and the structural motif with Pro14 between two Gly residues (P14G and P14GPG). AMPs with structural motifs containing glycine (Gly and GlyProGly), such as magainin 2 and ponericin G1, are observed to exert low hemolytic activity (Rodríguez et al., 2014). It was observed that proline to glycine substitution at the Pin2 [G] analog did not lead to the reduction of the hemolytic activity; however, the Pin2 [GPG] analog showed 5-fold reduction in hemolysis when compared with the native peptide Pin2 (Rodríguez et al., 2014). This demonstrates that the residue triad GlyProGly might have a fundamental role in hemolytic activity and can be altered to reduce it.

### Acidic Residue Modifications

Amino acids with a negatively charged R group at pH 7.0 include aspartic acid and glutamic acid, and generally are substituted by hydrophobic positively charged residues, due to the important role that these features play in the antimicrobial activity. La Salud Bea and collaborates (2017) studied the effect of lysine substitution by glutamic acid in the native peptide IsCT1 from the scorpion *O. madagascariensis*. The analog IsCT1E7, which presented a negative net charge of -1, did not show significant bacterial growth inhibition or apparent cytotoxicity at the conditions and concentrations tested. Controversially, substituting glutamic acid by lysine in Hp1404 from the scorpion *Heterometrus petersii* resulted in the analog named Hp1404-T1c, which showed 4-fold improvement in antimicrobial activity against *Pseudomonas aeruginosa* when compared with the native peptide (Kim et al., 2018). These achievements strengthen the hypothesis that positive charge has a considerable influence on these peptides’ mechanism of action.

### Hemolytic Activity

Although many AMPs show activity towards prokaryotic membranes, they may also interact and disturb eukaryotic membranes. The lack of specificity toward mammalian membranes is attributed not only to the lack of negatively charged lipids but also to the absence of a strong negative membrane potential in most mammalian membranes (Maturana et al., 2017). However, AMPs are able to interact with the erythrocyte surface by binding to sialic acid present in glycocalyx, glycoproteins, or glycosphingolipids (Hancock and Sahl, 2006). Increased hemolytic activity in most AMP analogs is commonly observed, and it can be associated with a higher net charge (Xie et al., 2017). La Salud Bea and contributors (2015) studied analogs from the native peptide BmKn1 from the scorpion *B. martensii* and argued that when modifications are made to enhance the positive net charge with lysine replacement, both antimicrobial and hemolytic activities increase.

The hemolytic activities of analogs from TsAP-1 and TsAP-2 were considerably affected by the enhanced net charge. By substituting four neutral amino acid residues by lysine, antimicrobial and hemolytic activities were dramatically affected (Table 2) (Guo et al., 2013). Therefore, it is evident that cytotoxicity against mammalian cells is a limiting factor for the therapeutic application of AMPs, but it is also clear that changes in sequence can overcome this challenge.

It is also worth pointing out that effective perturbation of the erythrocyte membranes is more dependent on high permeabilization efficiency than high lipidic affinity (Rodríguez et al., 2014; Maturana et al., 2017). Because hemolytic activity can be effectively reduced by decreasing amphipathicity (helix disturbance and reduced hydrophobic moment) and by decreasing the hydrophobic domain, these features are a powerful foundation to modulate AMP selectivity toward microbes (Rodríguez et al., 2014).

On the other hand, a study that did not modify peptides by increasing the charge but by replacing L-amino acids by D-amino acids with Pin2 used a D-diastereomer (D-Pin2) to assess whether it maintained the same antimicrobial activity as L-Pin2, and to reduce its hemolytic activity for human erythrocytes. Although the hydrophobic and secondary structure characteristics of L- and D-Pin2 were relatively similar, an important reduction in D-Pin2 hemolytic activity (30–40%) was achieved compared with that of L-Pin2. Moreover, the antimicrobial activity of D-Pin2 was reduced by half compared to that of L-Pin2 (Carmona et al., 2013).

In contrast to the observed hemolytic effects caused by increased net charge, Kim and contributors (2018) designed analogs of the scorpion peptide Hp1404. The analogs were generated through the substitution of neutral residues by lysine, which enhanced the net charge from +1 to +6. The modified AMPs showed lower hemolytic activity and lower cell toxicity than the native peptide, especially the analog Hp1404-T1e, which had a net charge of +6 and exhibited potent antimicrobial and antibiofilm activity against multiresistant *Pseudomonas aeruginosa* (MRPA) strains, and it was also functional in physiologic conditions. Modifications in Hp1404 demonstrate the potential use of the AMPs as efficient therapeutic agents and reinforce the possibility of designing safer peptides. Moreover, these results suggest that not only the net charge but also the primary structure and, consequently, the secondary conformation contributed to the hemolytic activity (Kim et al., 2018). Furthermore, *in vitro* assays provide an excellent starting point, but the antimicrobial field urgently needs *in vivo* studies to assess the toxicity and stability associated with the potential use of these peptides in therapy.
